# ncVarDB: a manually curated database for pathogenic non-coding variants and benign controls

**DOI:** 10.1093/database/baaa105

**Published:** 2020-12-01

**Authors:** Harry Biggs, Padmini Parthasarathy, Alexandra Gavryushkina, Paul P Gardner

**Affiliations:** Department of Biochemistry, University of Otago, PO Box 56, Dunedin 9054, New Zealand; Department of Biochemistry, University of Otago, PO Box 56, Dunedin 9054, New Zealand; Department of Biochemistry, University of Otago, PO Box 56, Dunedin 9054, New Zealand; Bio-Protection Research Centre, University of Otago, PO Box 56, Dunedin 9054, New Zealand; Department of Biochemistry, University of Otago, PO Box 56, Dunedin 9054, New Zealand; Bio-Protection Research Centre, University of Otago, PO Box 56, Dunedin 9054, New Zealand

## Abstract

Variants within the non-coding genome are frequently associated with phenotypes in genome-wide association studies. These non-coding regions may be involved in the regulation of gene expression, encode functional non-coding RNAs, or influence splicing and other cellular functions. We have curated a list of characterized non-coding human genome variants based on the published evidence that indicates phenotypic consequences of the variation. In order to minimize annotation errors, two curators have independently verified the supporting evidence for pathogenicity of each non-coding variant in the published literature. The database consists of 721 non-coding variants linked to the published literature describing the evidence of functional consequences. We have also sampled 7228 covariate-matched benign controls, that have a population frequency of over 5%, from the single nucleotide polymorphism database (dbSNP151) database. These were sampled controlling for potential confounding factors such as linkage with pathogenic variants, annotation type (untranslated region, intron, intergenic, etc.) and variant type (substitution or indel). The dataset presented here represents a curated repository, with a potential use for the training or evaluation of algorithms used in the prediction of non-coding variant functionality.

**Database URL**: https://github.com/Gardner-BinfLab/ncVarDB.

## Context

The advent of high-throughput sequencing has allowed the capture of millions of genome variants (1–3). The accessibility of genome variation data has spawned an industry of genome-wide association studies (GWAS), where genetic variation and phenotypic variation, such as disease susceptibility, are linked by statistical association tests (4). The combined results of GWAS have revealed that many variants that are linked to phenotypic consequences reside outside the protein-coding regions (5–7). These non-coding genetic variants can contribute to the phenotypic variation in a multitude of ways, including influencing alternative splicing and altering gene expression (8, 9). The study of non-coding variation has been hampered by a lack of molecular and computation tools for analysing the consequences of these variants (6).

Non-coding variants may influence gene expression and splicing, be functional non-coding RNAs (ncRNAs) or, as is frequently the case, be of unknown importance (6, 10). The experimental validation of every non-coding variant discovered *in silico* is currently not feasible; therefore, computational methods that can prioritize variants that are likely to have functional impacts are a research priority (11–13). These computational methods require reliable training and evaluation data to learn features that are indicative of a functional impact. Many of the existing non-coding variant annotation tools have been built using training and evaluation datasets constructed from public repositories such as ClinVar (14).

Recent benchmarks show that while these tools perform well with ClinVar variants (e.g. area under the curve [AUC] values >0.95), the tools do not perform as well against other databases such as the Catalogue Of Somatic Mutations In Cancer (15) (AUC values <0.78) (16, 17). The functional probing of saturation mutation of disease-associated gene promoters and enhancers has provided further independent data for evaluating the accuracy of non-coding pathogenicity prediction tools. This approach has also highlighted the relatively poor predictive performance of these methods (AUC values between 0.53 and 0.75) (18). The benchmarking of pathogenicity prediction tools for protein coding variants has highlighted the issue of overtraining of methods on the evaluation data, which is likely to also be a problem for non-coding methods (19).

Errors in biological databases are an ever-present concern for database curators, which impact the training, development and evaluation of different methods (20). The accuracy of functional annotations of genes (21, 22), taxonomic origins of sequences (23), and variant classifications (24) can unduly influence the conclusions of research that relies on accurate database information. This issue has led to calls to allow researchers to directly edit entries in leading sequence databases in order to correct errors (25, 26).

In order to partially address the issues of overtraining upon existing databases and minimize the number of errors in human non-coding variant classification databases, we have produced a manually curated variant classification database (ncVarDB). Two separate data curators have curated non-coding pathogenic variants directly from the published literature and from public data repositories. The current database release contains 721 pathogenic variants and 7228 the single nucleotide polymorphism database (dbSNP)-derived benign variants.

## Data description

Two datasets were generated, one containing pathogenic variants supported by the published literature and one containing presumed benign variants. Multiple publicly available data repositories were used in the curation of these datasets. The pathogenic dataset was generated using the October 2019 release of ClinVar (14) and OMIM (27), also accessed between April and October 2019.

We selected non-coding variants from the ClinVar database (see Supplementary Data for details) and assessed the cited (by ClinVar) literature for confirmation of each variant. In cases where there was no citation available for the entry, the entry was excluded. In cases where the citation either did not contain genomic position information or contained information for a different mutation, the variant was also excluded.

To identify the well-characterized disease-associated variants that lie within non-coding genes, Online Mendelian Inheritance in Man (OMIM) was mined for ncRNA variants. We manually identified ncRNA variants and again confirmed that the pathogenicity of each variant was correctly mentioned in the citation. With further literature searches, additional three variants were included, such as variants in RMRP, the variation that may cause cartilage hair hypoplasia. After these methods, 721 pathogenic variants were kept for use.

**Figure 1.**
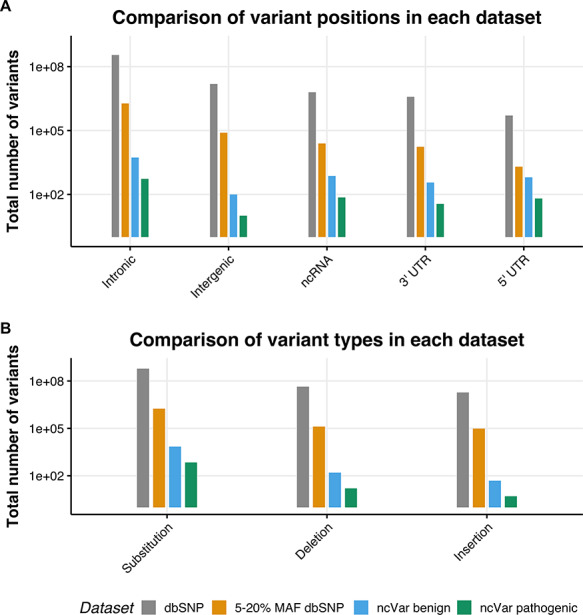
The location and single-nucleotide polymorphism (SNP) types of ncVarDB variants in comparison to variants from the dbSNP database. A comparison of the variant positions and the type of variants in every SNP in dbSNP dataset excluding variants from alternate contigs (dbSNP), every non-coding SNP with a MAF between 5 and 20% (5–20% MAF dbSNP), the ncVar benign dataset and the ncVar pathogenic dataset. (A) A comparison of the frequency of genomic positions of variants present in each dataset. Positions are based on the genomic notation submitted with the variant in either dbSNP or ClinVar. (B) A comparison of the frequency of variant types for each dataset. Variant types have been simplified to three types to avoid type expansion.

We generated a set of benign non-coding variants from variants in the dbSNP151 database (1) using the University of California, Santa Cruz (UCSC) table browser tool (28, 29). Variants with a minor allele frequency (MAF) between 5 and 20% are likely to be benign, as stated in the 2015 American College of Medical Genetics guidelines (30). Any variant with a MAF between 5 and 20% in the entire dbSNP151 database was selected, with no alternate chromosomes included. This set was then randomly sampled with 10 benign variants being sampled for each pathogenic variant, the proportions of variant positions (e.g. intergenic, untranslated region [UTR] or intronic) and variant types (e.g. substitution, insertion or deletion) were kept the same. In order to control for linkage, no variant within 30 kb of a ncVarDB pathogenic variant was selected. These are estimated to have a <1% chance of being in linkage with the non-coding pathogenic variants (31). A comparison of the datasets is provided in Figure 1.

A potential confounding factor in this database is the lack of pathogenic variants that lie in intergenic regions. There is a low number of intergenic variants in the ncVarDB pathogenic dataset in comparison to other variant positions. Some potential reasons for this are discovery bias or verification bias. Because the original variant discovery was performed using database searches in ClinVar and OMIM, without searching specifically for intergenic regions, the variants lying in those regions may have not been captured by the original variant screening process. Another possibility is that as the variants in the database have been biologically validated for phenotypic changes, these variants are more likely to be in genic regions as these regions are traditionally of more interest to researchers studying genetic diseases.

The control benign dataset has been assembled automatically and not manually curated. Rare errors can occur in the benign dataset due to errors or ambiguities in the dbSNP database (see Supplementary data). There are very few variants on the mitochondrial chromosome in the dbSNP database that have a known molecular function and MAF value; as a result, ncVar benign dataset by chance does not contain variants on the mitochondrial chromosome.

The two datasets contain the following:

ID: An ID for this database

Genome: The genome that the variant was found in

Chr: The chromosome the variant is in

Pos: The starting position of the variant (referring to the first affected nucleotide). In case of a substitution (or a deletion), the starting position is the first nucleotide in the substituted (or deleted) sequence. In case of an insertion the starting position is the position of the nucleotide after which a new sequence is inserted.

Ref: The reference genome sequence

Alt: The variant sequence

Mutation_type: the type of mutation of the variant (substitution, insertion, deletion)

Mutation_position: The genomic position of the variant (intronic, 5utr, 3utr, ncRNA, intergenic)

MAF: The frequency of the minor allele (Alt)

X_ref: Any ID’s from other databases e.g. dbSNP [REF] ClinVar [REF], OMIM [REF], Literature

The pathogenic dataset has extra two columns:

Pubmed_ID: A pubmed identifier that relates to literature that confirms the pathogenicity of the variant

Phenotype: The phenotype associated with the variant (sourced from XXXX)

The database can be found in ncVarDB (32).

## Data analysis

We classified ncVarDB variants using popular software tools: Functional Analysis Through Hidden Markov Models with an eXtended Feature set (FATHMM-XF) (33), Combined Annotation Dependent Depletion (CADD) v1.4 (11, 34) and Deleterious Annotation of genetic variants using Neural Networks (DANN) (12). FATHMM-XF and CADD v1.4 use statistical learning techniques (a support vector machine and a logistic regression model, respectively) to assign scores to variants based on conservation scores and other genomic features. Although there are differences in classification methods and the sets of features in the two software tools, the main difference is in the training sets. FATHMM-XF used previously identified pathogenic and benign variants from public databases. The training set for CADD consists of high frequency derived alleles in the human genome (compared to the inferred genome of the human-ape ancestor) as a ‘proxy-benign’ (neutral) group and simulated, free of selective pressure, variants as a ‘proxy-pathogenic’ group. DANN uses the same training set as CADD but uses a deep neural network algorithm for the classification.

The online FATHMM-XF tool was used for scoring our pathogenic and benign variants. This programme does not score insertions, deletions and more than one nucleotide long substitutions. It does not score variants on chromosomes X, Y and M. Excluding these variants and several additional variants that caused an error (see Supplementary data), we performed a receiver operating characteristic (ROC) curve analysis on 569 (79% of all ncVarDB pathogenic variants) pathogenic and 6823 (94% of all ncVarDB benign variants) benign ncVarDB variants that received FATHMM-XF score.

We used online CADD scoring implementation with raw scores for ROC curve analysis. CADD does not score variants on chromosome M. In total, 656 (91%) pathogenic and 7228 benign variants (all variants) were scored by CADD.

For DANN analysis, we downloaded precomputed scores provided by the authors for single nucleotide variants. DANN does not score variants on chromosomes Y and M. The positions of the DANN scored variants are provided relative to GRCH37/hg19 assembly. We converted positions of ncVarDB variants to positions relative to GRCH37/hg19 assembly using the UCSC hgLiftOver tool (29). Due to conversion errors, several variants were further excluded (see Supplementary data), which resulted in 633 (88%) pathogenic and 6989 (97%) benign DANN scored variants.

**Figure 2.**
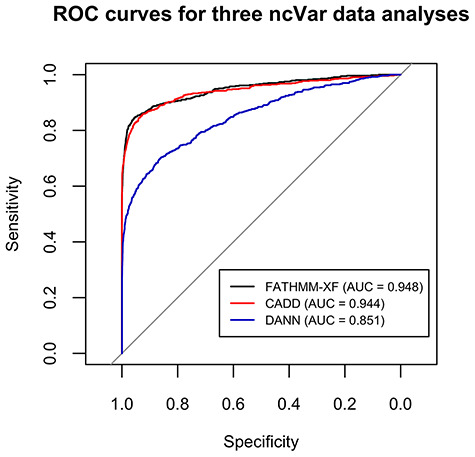
ROC curves for the classification analyses of the ncVar dataset by three different software tools: FATHMM-XF, CADD v1.4 and DANN. FATHMM-XF and CADD predict the pathogenicity of the ncVarDB variants with noticeably higher specificity and sensitivity than DANN. Overall good performance of all three tools additionally validates the ncVar dataset.

FATHMM-XF and CADD performed well on the ncVar dataset with AUCs of 0.948 and 0.944, respectively (Figure 2). The AUC for DANN analysis was 0.851 (Figure 2). Several previous comparisons of scoring methods showed closer AUC values (up to 0.06 difference) for CADD and DANN analyses (12, 34, 35).

The accurate classification of the ncVar dataset by the three popular scoring tools additionally validates the dataset. These analyses are also an example of the potential use of the ncVar dataset for evaluation of the scoring method performance.

## Data validation and quality control

To ensure a high level of fidelity, each variant was inspected by two different data curators. Each variant in this database contains a link to a PubMed article that was used to verify that variant.

## Re-use potential

This database contains a test set and a control set containing pathogenic variants and benign variants. This has a wide range of potential uses, such as training algorithms for predicting a non-coding variant functionality.

## Supplementary Material

baaa105_Supp

## Data Availability

All entries in these datasets link back to several publicly available data repositories.

## Data description

Two TSV files and two VCF files containing variant information. Pathogenic variant information has been manually curated.
