# Supplementary material for: ncVarDB: a manually curated database for pathogenic non-coding variants and benign controls
Source: Database (Oxford). 2020 Dec 1;2020:baaa105. doi: 10.1093/database/baaa105 (PMC7706182; doi:10.1093/database/baaa105)
Supplement: baaa105_Supp [file baaa105_supp.zip › Supplementary_Information.docx]

**Supplementary Information**

ClinVar and OMIM search parameters:

Clinvar was searched using the search parameters:

- *(((((("clinsig likely path"[Filter] OR "clinsig pathogenic"[Filter])))) AND ((("2kb upstream variant"[Molecular consequence] OR "3 prime utr variant"[Molecular consequence] OR "5 prime utr variant"[Molecular consequence] OR "500b downstream variant"[Molecular consequence] OR "intergenic variant"[Molecular consequence] OR "intron variant"[Molecular consequence])))) AND "single nucleotide variant"[Type of variation])*

OMIM was mined for ncRNA variants using the search parameters:

- *“tRNA -synthetase -transferase -methyltransferase -NUCLEOTIDYLTRANSFERASE” OR “miRNA” OR “snoRNA” OR “lncRNA” OR "RNAse P"*

Benign dataset search parameters:

To download all non-coding variants from dbSNP dataset we used UCSC table browser setting “Common SNPs(151)” track and filter:

*not (snp151Common.chrom like '%\\_%') and not (FIND_IN_SET('cds-indel', snp151Common.func)>0 OR FIND_IN_SET('frameshift', snp151Common.func)>0 OR FIND_IN_SET('stop-loss', snp151Common.func)>0 OR FIND_IN_SET('missense', snp151Common.func)>0 OR FIND_IN_SET('nonsense', snp151Common.func)>0 OR FIND_IN_SET('coding-synon', snp151Common.func)>0 OR FIND_IN_SET('unknown', snp151Common.func)>0 )*

Possible errors in benign ncVar dataset:

A small number of benign mutations might not be correct due to the ambiguous data in the data file downloaded from dbSNP using UCSC table browser. For the complement strand (“-” in the dbsnp151 data file), the reference allele is always in the forward format but the possible alleles could be in either format. To identify which strand is used for the list of possible alleles we took the major allele (the allele with the highest reported frequency in the datafile) and compared to the reference. If the major allele is the same as the reference we conclude the possible alleles are in the forward format. If the major allele is the complement of the reference then we conclude that the possible alleles are in the reverse format. Thus, in rare cases where the major allele does not correspond to the reference but occurs to be the same as the complement of the reference, the entry in ncVar benign dataset will not be correct.

Scoring errors:

No prediction was found by FATHMM-XF for two pathogenic variants and eleven benign variants. Six pathogenic and nine benign variants were classified as coding by FATHMM-XF tool and were not included in the ROC curve analysis.

For DANN analysis, three benign variants failed to convert from positions relative to GRCH38/hg38 to positions relative to GRCH37/hg19 and were excluded from the analysis. Three pathogenic and 17 more benign variants were excluded due to inconsistencies between the two assemblies
